# Demographics, Lifestyle, Comorbidities, Prediabetes, and Mortality

**DOI:** 10.1001/jamanetworkopen.2025.26219

**Published:** 2025-08-07

**Authors:** Obinna Ekwunife, Xuemeng Wang, Raphael Fraser, Jennifer A. Campbell, Rebekah J. Walker, David Jacobs, Leonard E. Egede

**Affiliations:** 1 Division of Population Health, Department of Medicine, Jacobs School of Medicine and Biomedical Sciences, University at Buffalo, Buffalo, New York; 2 Division of Outcomes and Practice Advancement, Department of Pharmacy Practice, School of Pharmacy, University at Buffalo, Buffalo, New York

## Abstract

This cohort study examines the complexities of associations between prediabetes and mortality when adjusted for modifying factors, include demographics, lifestyle, and comorbidities.

## Introduction

In addition to increasing the risk of developing type 2 diabetes,^1,2^ prediabetes is also linked to increased cardiovascular disease risk^3^ and elevated all-cause and cause-specific mortality.^4,5^ However, the association between prediabetes and mortality remains complex, particularly when accounting for factors such as demographics, lifestyle, and comorbidities. To better understand how these factors affect the association, this study examines their modifying effect in a US adult population with prediabetes. Associations were assessed by adjusting for 3 levels of factors: demographic factors alone, demographic and lifestyle factors, and demographic, lifestyle, and comorbidity factors.

## Methods

Following STROBE guidelines, this cohort study used data from the National Center for Health Statistics linked to the National Death Index mortality follow-up for individuals who participated in the National Health and Nutrition Examination Survey (NHANES) from 1999 through 2018.^6^ Adults 20 years or older who completed both the interview and physical examination, had valid mortality data, and participated in survey cycles from 2005 to 2018 were included. Prediabetes was defined by self-report or hemoglobin A^1c^ level (5.7%-6.4%) using NHANES data. Covariates included demographics, lifestyle, and comorbidities. Race and ethnicity were self-reported during NHANES interviews, and participants were categorized as non-Hispanic Black, non-Hispanic White, or other (due to limited sample size). Multivariable Cox proportional hazards models were used to assess associations and adjust for potential confounders. We also conducted stratified analyses by age group and race and ethnicity to examine possible modification effect. Analyses used weighted data and R, version 4.4.1 (R Foundation for Statistical Computing), with significance set at *P* < .05. This retrospective analysis of deidentified data did not require institutional review board approval or patient consent, in accordance with 45 CFR §46.102(e).

## Results

Of the 38 093 respondents, 9971 (26.2%), representing more than 51 million US adults, had prediabetes. Table 1 shows weighted demographics of these individuals. Most were female and aged 20 to 54 years. Prediabetes was initially associated with mortality (hazard ratio [HR], 1.58; 95% CI, 1.43-1.74), but lost significance in the fully adjusted model (HR, 1.04; 95% CI, 0.92-1.18) (Table 2). Significant interactions were observed between prediabetes and age group and race and ethnicity. Stratified Cox models found that prediabetes was statistically significantly associated with mortality only among adults aged 20 to 54 years (HR, 1.64; 95% CI, 1.24-2.17) (Table 2). No significant associations were found among racial and ethnic groups.

**Table 1.** Weighted Sample Characteristics for Adults Overall and Stratified by Prediabetes Status (2005-2018)

**Characteristic**	All respondents, No. (weighted %) (N = 38 093)^a^	With prediabetes, No. (weighted %) (n = 9971)^a^	Deaths among those with prediabetes, No.^a^	Without prediabetes, No. (weighted %) (n = 28 122)^a^	Deaths among those without prediabetes, No.^a^
**Demographic**					
Age, y^b^					
20-54	22 492 (65.1)	4374 (46.8)	126	18 118 (70.6)	416
55-74	11 426 (27.1)	4076 (40.6)	437	7350 (23.1)	1223
≥75	4175 (7.8)	1521 (12.6)	666	2654 (6.3)	1343
Sex					
Male	18 427 (48.1)	4801 (46.3)	NA	13 626 (48.7)	NA
Female	19 666 (51.9)	5170 (53.7)	NA	14 496 (51.3)	NA
Race and ethnicity^b^					
Non-Hispanic Black	8295 (11.4)	2559 (14.6)	227	5736 (10.4)	677
Non-Hispanic White	15 891 (66.7)	3755 (62.7)	796	12 136 (67.9)	1745
Other^c^	13 907 (21.9)	3657 (22.7)	206	10 250 (21.6)	560
Marital status					
Not married	18 526 (44.9)	4611 (41.9)	NA	13 915 (45.8)	NA
Married	19 542 (55.1)	5353 (58.1)	NA	14 189 (54.2)	NA
**Lifestyle**					
Smoking status					
Non-smoker	21 127 (55.2)	5253 (51.7)	NA	15 874 (56.3)	NA
Former smoker	9115 (24.5)	2651 (27.3)	NA	6464 (23.6)	NA
Current smoker	7822 (20.3)	2057 (21.0)	NA	5765 (20.1)	NA
≥12 Drinks in past y	22 933 (82.3)	5703 (77.2)	NA	17 230 (83.9)	NA
**Comorbities**					
Diabetes	4942 (9.6)	0	NA	4942 (12.5)	NA
Hypertension	13 669 (31.8)	4500 (43.4)	NA	9169 (28.3)	NA
Heart disease	3291 (6.9)	1062 (10.0)	NA	2229 (5.9)	NA
Stroke	1505 (2.9)	436 (3.7)	NA	1069 (2.7)	NA
Cancer	3620 (10.1)	1141 (13.2)	NA	2479 (9.1)	NA
Body mass index, mean (SD)^d^	29 (7)	29 (7)	NA	31 (7)	NA
**Mortality status**					
Alive	33 882 (91.9)	8742 (89.6)	NA	25 140 (92.6)	NA
Deceased	4211 (8.1)	1229 (10.4)	1229	2982 (7.4)	2982

Abbreviations: NA, not applicable.

^a^ Numbers are unweighted. The percentages reflect weighted proportions representative of the US civilian noninstitutionalized population. The weighted totals are: (1) overall population: 223 888 859; (2) with prediabetes: 51 836 778; and (3) without prediabetes: 172 052 081.

^b^ Distribution of deaths across age groups and race and ethnicity was added since significant interactions were observed between prediabetes and both age group and race and ethnicity. The mortality data allows for better evaluation of whether the observed age-specific association reflects underlying difference in statistical power. Mortality counts reflect unweighted numbers of deaths by category.

^c^ Other includes individuals identified as Asian, Mexican American, multiracial, other Hispanic, or other race not classified as non-Hispanic White or non-Hispanic Black, according to National Health and Nutrition Examination Survey categorization.

^d^ Calculated as weight in kilograms divided by height in meters squared.

**Table 2.** Cox Proportional Hazards Models Examining the Association Between Prediabetes and Mortality, Overall and Stratified by Age and Race and Ethnicity

Model^a^	HR (95% CI)	*P* value
Overall		
Unadjusted	1.58 (1.43-1.74)	<.001
Adjusted for demographics	0.88 (0.80-0.98)	.02
Adjusted for demographics and lifestyle factors	0.92 (0.82-1.04)	.17
Adjusted for demographics, lifestyle factors, and comorbidities (fully adjusted)	1.05 (0.92-1.19)	.47
Stratified by age group		
20-54 y	1.68 (1.25-2.20)	<.001
55-74 y	0.87 (0.69-1.09)	.22
≥75 y	0.97 (0.83-1.12)	.66
Stratified by race and ethnicity		
Non-Hispanic Black	1.02 (0.80-1.30)	.86
Non-Hispanic White	1.06 (0.91-1.23)	.45
Other^b^	0.81 (0.56-1.19)	.29

Abbreviation: HR, hazard ratio.

^a^ Results are for respondents with prediabetes; the reference group was those without prediabetes.

^b^ Other includes individuals identified as Mexican American, Other Hispanic, Asian, multiracial, or other race not classified as non-Hispanic White or non-Hispanic Black, according to National Health and Nutrition Examination Survey categorization.

## Discussion

Using NHANES data linked to mortality records, this cohort study found that while unadjusted models showed a significant association as reported by several studies,^4,5^ adjusting for demographic, lifestyle factors, and comorbidities attenuated this finding (see Supplement 1). Stratified analyses revealed that prediabetes was significantly associated with mortality only among younger adults (age 20-54 years), highlighting the importance of age-specific interventions. Lifestyle behaviors, limited health care access, and life stage challenges may contribute to the increased mortality risk in younger adults. Early-onset health problems in this group may also reflect stronger genetic predispositions, leading to more rapid disease progression and more severe health outcomes. These findings underscore the need for tailored diabetes prevention programs targeting young adults—such as flexible, virtual, and peer-led options—to increase accessibility and engagement. Routine screening and timely referrals to age-appropriate programs are essential. Although the use of nationally representative NHANES data strengthens the study, limitations include its cross-sectional design, potential self-report bias, lack of longitudinal tracking, and the inability to infer causality due to its observational nature. Future research should focus on longitudinal studies and targeted interventions to reduce mortality among young adults with prediabetes. Early intervention is key to preventing disease progression and improving long-term health outcomes.

## Notes

Supplement 1. **eAppendix.** Supplementary Methods
eReferences.

Supplement 2.
Data Sharing Statement
