# Supplementary material for: Demographics, Lifestyle, Comorbidities, Prediabetes, and Mortality
Source: JAMA Netw Open. 2025 Aug 7;8(8):e2526219. doi: 10.1001/jamanetworkopen.2025.26219 (PMC12332616; doi:10.1001/jamanetworkopen.2025.26219)
Supplement: Supplement 1. — eAppendix. Supplementary Methods eReferences. [file jamanetwopen-e2526219-s001.pdf]

Supplemental Online Content

Ekwunife O, Wang X, Fraser R, et al. Demographics, lifestyle, Comorbidities, Prediabetes, and Mortality.. *JAMA Netw Open.* 2025;8(8):e2526219. doi:10.1001/jamanetworkopen.2025.26219

Supplement 1. eAppendix. Supplementary Methods

eReferences.

This supplemental material has been provided by the authors to give readers additional information about their work.

© 2025 Ekwunife O et al. *JAMA Network Open*

eAppendix. Supplementary Methods

Description of Dataset

This study used National Center for Health Statistics data linked to the National Death Index (NDI) mortality follow-up data for individuals who participated in the National Health and Nutrition Examination Survey (NHANES) from 1999 through 2018.[1,2]
his linkage enables researchers to analyze mortality outcomes by connecting NHANES survey data with verified death records from the NDI.[12] The public-use linked mortality files offer information on mortality status and cause of death for NHANES participants over this period. This linkage facilitates a robust examination of associations between health behaviors, conditions, and various health outcomes and mortality risks in the U.S. population. Mortality follow-up is conducted through a probabilistic matching process between NHANES records and NDI data.[1] The linked data cover death outcomes and dates, causes of death, and underlying disease conditions coded according to the International Classification of Diseases.[3]
Variables

Mortality outcome

Mortality outcome was obtained from the National Center for Health Statistics public-used linked mortality file through 2018.[1] Each eligible survey participant for mortality follow-up was categorized as either assumed alive or assumed deceased based on NDI status. Mortality follow-up was calculated from the interview date to December 31, 2019.

Primary independent variable

Prediabetes was defined as either self-reported prediabetes or prediabetes based on HbA1c values, using three variables from the NHANES dataset: self-reported prediabetes, self-reported diabetes, and A1c levels. Participants who answered "yes" to the question *"Have you ever been told by a doctor or other health professional that you have any of the following: prediabetes, impaired fasting glucose, impaired glucose tolerance, borderline diabetes, or that your blood sugar is higher than normal but not high enough to be called diabetes or sugar diabetes?"* were categorized as having self-reported prediabetes. Participants with an HbA1c value between 5.7% and 6.4% who answered "no" or "borderline" to the question, *"Have you ever been told by a doctor or health professional that you have diabetes or sugar diabetes?"* were categorized as having prediabetes based on HbA1c values. Participants meeting either

the self-report or HbA1c criteria were classified as "yes" for prediabetes, while those who did not were classified as "no".

Covariates

Covariates were classified into 3 sections: demographic, lifestyle and comorbidity factors. Demographic factors included age group (20-54 yrs, 55-74 yrs 75+ yrs), race/ethnicity (non-Hispanic White, non-Hispanic Black and other), marital status (married, not married) and sex (male, female). Lifestyle factors included smoking status (non-smoker, former smoker and current smoker) and drinking status (yes/no). Comorbidity included self-reported diabetes, hypertension, heart disease (congestive heart failure/ coronary heart disease/ coronary heart disease/ (angina/angina pectoris)/ heart attack), stroke, cancer (each coded as yes/no) or BMI (continuous variable).

Statistical Analysis

Baseline characteristics of participants were summarized using weighted frequencies and percentages, reflecting the complex survey design and sampling weights of NHANES. In addition, all analyses were weighted using the complex survey design and sampling weights to provide estimates reflective of the US adult population.[2] Cox proportional hazards regression models were used to evaluate the association between prediabetes and all-cause mortality, adjusting for demographic, lifestyle, and comorbidity factors. A sequence of four models were run, each introducing an additional block of covariates to assess how various factors influenced mortality risk. First, an unadjusted model was run. Second, the model was adjusted for demographic factors (age, race/ethnicity, marital status, and sex). Third, the model was further adjusted for lifestyle factors (smoking status and drinking status). Finally, the model was adjusted for comorbidity factors. Model assumptions were assessed using Schoenfeld residuals, and no significant violations of the proportional hazards assumption were identified. Cox interaction models were run to test whether the effect of prediabetes on all-cause mortality differed by three demographic factors (sex, age, and race/ethnicity) individually. The interaction term for sex was not significant (p-value =0.46). Interaction terms for age and race/ethnicity were significant (age: p-value <0.001 for both 55-74 yrs and 75+ yrs interactions; Race/ethnicity: p-value<0.001 for non-Hispanic Black interaction and 0.006 for other racial interaction). Then cox stratified models were conducted for age and race/ethnicity groups adjusting for demographic, lifestyle, and comorbidity factors. An unadjusted Kaplan-Meier survival curve was generated to compare survival probabilities over time between participants with and without

prediabetes and additional unadjusted Kaplan-Meier survival curves were also created for prediabetes yes only cohort to compare the survival rates between age and race/ethnicity groups. Statistical significance was defined as a two-sided alpha level of 0.05. All analyses were conducted using R software, version 4.4.1.[4]

eReferences

1. Centers for Disease Control and Prevention. National Death Index [Internet]. 2024 Dec 23 [cited 2024 Nov 21]. Available from: https://www.cdc.gov/nchs/ndi/index.html
2. Centers for Disease Control and Prevention. National Health and Nutrition Examination Survey [Internet]. 2025 Jan 15 [cited 2024 Nov 21]. Available from: https://www.cdc.gov/nchs/nhanes/index.html
3. World Health Organization. International Classification of Diseases (ICD) [Internet]. 2019 [cited 2024 Nov 21]. Available from: https://www.who.int/standards/classifications/classification-of-diseases
4. R Core Team. R: The R Project for Statistical Computing [Internet]. Vienna, Austria: R Foundation for Statistical Computing; [cited 2024 Nov 21]. Available from: https://www.r-project.org/
