# Supplementary material for: Demographics, Lifestyle, Comorbidities, Prediabetes, and Mortality
Source: JAMA Netw Open. 2025 Aug 7;8(8):e2526219. doi: 10.1001/jamanetworkopen.2025.26219 (PMC12332616; doi:10.1001/jamanetworkopen.2025.26219)
Supplement: Supplement 2. — Data Sharing Statement [file jamanetwopen-e2526219-s002.pdf]

Data Sharing Statement

Ekwunife. Demographics, Lifestyle, Comorbidities, Prediabetes, and Mortality. *JAMA Netw Open*. Published August 07, 2025. doi:10.1001/jamanetworkopen.2025.26219

Data
Data available: No

Additional Information
Explanation for why data not available: The dataset supporting the conclusions of this article are publicly available from the Centers for Disease Control and Prevention website: https://www.cdc.gov/nchs/ndi/index.html; https://www.cdc.gov/nchs/nhanes/index.html
